# Spatio-temporal variation in tuberculosis incidence and risk factors for the disease in a region of unbalanced socio-economic development

**DOI:** 10.1186/s12889-021-11833-2

**Published:** 2021-10-09

**Authors:** Li Wang, Chengdong Xu, Maogui Hu, Jiajun Qiao, Wei Chen, Tao Li, Songbo Qian, Mingtao Yan

**Affiliations:** 1 College of geography and environmental science, Henan University, KaiFeng, 475001 China; 2 Key Laboratory of Geospatial Technology for the Middle and Lower Yellow River Regions (Henan University), Ministry of Education, KaiFeng, 475001 China; 3 State Key Laboratory of Resources and Environmental Information System, Institute of Geographic Science and Natural Resource Research, Chinese Academy of Sciences, Beijing, 100101 China; 4 University of Chinese Academy of Sciences, Beijing, 100049 China; 5 Chinese Center for Disease Control and Prevention, Beijing, 102206 China; 6 Changchun University of Science and Technology, Changchun, 130022 China

**Keywords:** Tuberculosis, Aging population, GeoDetector, Bayesian space–time hierarchy model, Socio-economic status, Disease risk factors

## Abstract

**Background:**

Previous research pointed to a close relationship between the incidence of tuberculosis (TB) in aging populations and socio-economic conditions, however there has been lack of studies focused on a region of unbalanced socio-economic development. The aim of this paper is to explore the spatio-temporal variation in TB incidence and examine risk determinants of the disease among aging populations in a typical region.

**Methods:**

Data on TB-registered cases between 2009 and 2014, in addition to social-economic factors, were collected for each district/county in Beijing, Tianjin and Hebei, a region characterized by an aging population and disparities in social-economic development. A Bayesian space–time hierarchy model (BSTHM) was used to reveal spatio-temporal variation in the incidence of TB among the elderly in this region between 2009 to 2014. GeoDetector was applied to measure the determinant power (*q* statistic) of risk factors for TB among the elderly.

**Results:**

The incidence of TB among the elderly exhibited geographical spatial heterogeneity, with a higher incidence in underdeveloped rural areas compared with that in urban areas. Hotspots of TB incidence risk among the elderly were mostly located in north-eastern and southern areas in the study region, far from metropolitan areas. Areas with low risk were distributed mainly in the Beijing-Tianjin metropolitan areas. Social-economic factors had a non-linear influence on elderly TB incidence, with the dominant factors among rural populations being income (*q* = 0.20) and medical conditions (*q* = 0.17). These factors had a non-linear interactive effect on the incidence of TB among the elderly, with medical conditions and the level of economic development having the strongest effect (*q* = 0.54).

**Conclusions:**

The findings explain spatio-temporal variation in TB incidence and risk determinants of elderly TB in the presence of disparities in social-economic development. High-risk zones were located mainly in rural areas, far from metropolitan centres. Medical conditions and the economic development level were significantly associated with elderly TB incidence, and these factors had a non-linear interactive effect on elderly TB incidence. The findings can help to optimize the allocation of health resources and to control TB transmission in the aging population in this region.

## Background

Tuberculosis (TB) is an airborne infectious disease caused by the *Mycobacterium tuberculosis* complex; only pulmonary TB is contagious [1]. It is a serious and persistent infectious disease worldwide, which poses a threat to human health, resulting in approximately 10 million illnesses every year [2]. According to World Health Organization (WHO) report, TB is ranked as one of the top 10 leading causes of death from a single infectious agent worldwide [3]. The elderly population carries the majority of the TB burden [4].

TB has been considered a global public health emergency for the past decades, and much progress in reducing the incidence of TB has been made, however, no country has yet eliminated TB [5]. Although the TB incidence rate is falling worldwide, with a decrease of 9% reported between 2015 and 2020, it has yet to reach the first milestone of the End TB Strategy (i.e. a 20% reduction in this period) [3]. TB remains a major public health problem in China, where there are nearly 830,000 new cases each year [3].

The demographic structure of a population is a key determinant of infectious disease spread [6]. In socially underprivileged areas, elderly individuals have an increased risk of contracting TB [7, 8]. The TB burden in the aging population remains a clinical and epidemiological challenge.

According to some research, regional TB incidence is closely associated with poverty, poor living conditions and human agglomeration [9]. Poverty may affect susceptibility to infections and diseases through indirect effects of poor nutrition or depressed immune status [10]. Based on current evidence, income, education, occupation and population density account for most health inequalities within countries [11–15]. Furthermore, unbalanced regional development, with resulting consequences for socio-economic conditions, plays an important role in the incidence of TB [12].

Using various spatial statistical methods, previous research confirmed that the risk of TB transmission is spatially heterogeneous. These methods included Moran’s I and spatial panel data models [16], Kernel density estimation [17], spatial regression models [18] and geographically weighted regression models [19]. However, as these studies analysed only spatial aspects of TB transmission, they cannot reveal spatio-temporal variation in TB incidence and risk determinants of the disease in regions with unbalanced socio-economic development.

There were two primary aims of the present study: The first aim was to investigate spatio-temporal variation in the incidence of TB among individuals older than 65 years in the Beijing-Tianjin-Hebei urban agglomeration, which is a region with a high proportion of aging households and socio-economic disparities. The second aim was to investigate whether there was a non-linear association between the risk of TB in this aging population and socio-economic factors.

## Materials

### Study region

The study region was the North China Plain and consisted of the Beijing Municipality, Tianjin Municipality and Hebei Province. This region shows spatial heterogeneity in economic development. Beijing is the capital of China, Tianjin is one of a number of municipalities directly under the control of central government. Two neighbouring cities constitute a large metropolis. Surrounding the two cities is Hebei Province.

Economic development in the region is unbalanced, with many of the poorest counties in the country during the study period located in Hebei Province. In addition, aging of the population is becoming a growing concern in the study region, as the proportion of the population aged older than 65 years in Beijing, Tianjin and Hebei is 8.72, 8.53 and 8.69%, respectively. This index is an important international criterion used to measure the degree of population aging in a country or a region, with values over 7% constituting an aging society.

### Data

**Fig. 1:**
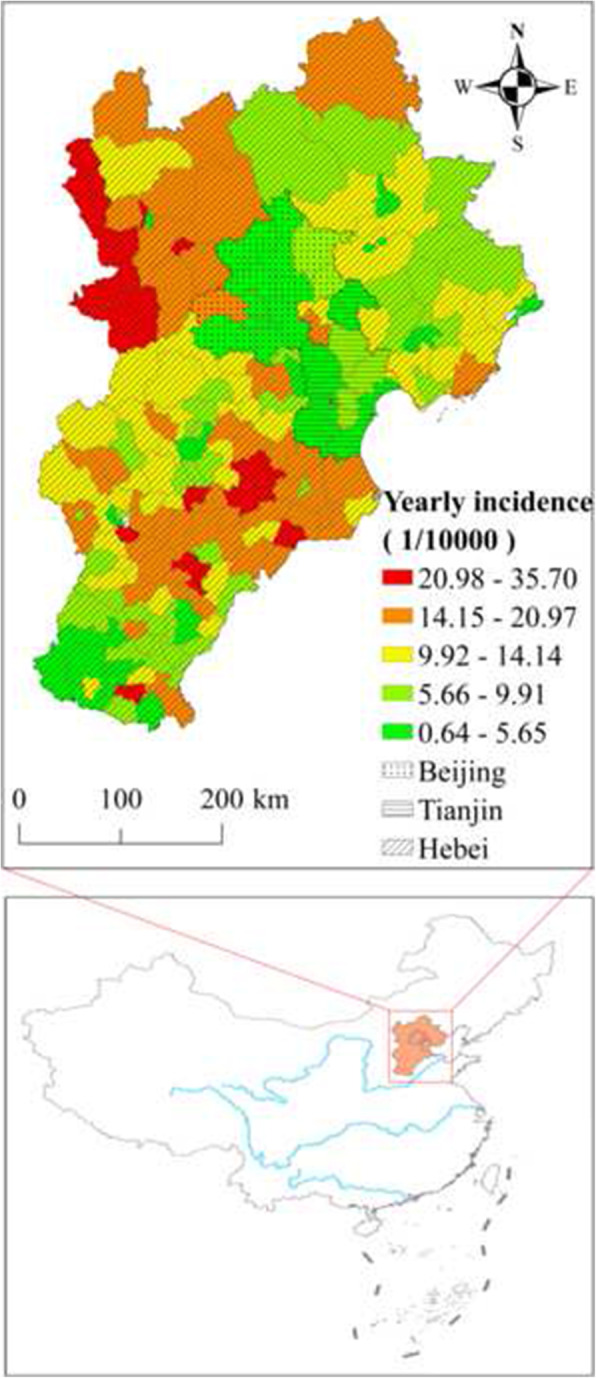
Geographic distribution of the Beijing–Tianjin–Hebei region in China and average annual tuberculosis incidence in 2009–2014. This figure was drawn by the authors using ArcGIS 10.2 software

In this study, data on TB cases in Beijing, Tianjin and Hebei were obtained from the Chinese Center for Disease Control and Prevention, which is a national real-time surveillance system, and the Notifiable Disease Reporting System, which covers all districts and counties in the study region. In total, data on 48,213 TB patients older than 65 years were collected. The study period was 2009–2014. Figure 1 shows the geographic distribution of TB incidence in the Beijing–Tianjin–Hebei region in China.

Data on potential factors related to TB in the region in 2009–2014 were collected from the statistical yearbook, which includes information on Gross Domestic Product (GDP) per capita, population density, industrial structure, urban and rural household income and number of beds in medical facilities (Table 1).

**Table 1:** Descriptive characteristics for various risk factors and TB incidence

Variables	Abbreviation	Minimum	Maximum	Mean	median	Standard deviation
TB incidence (1/10^5^)	*y*	10.3	1905.1	411.6	385.7	231.0
Per capita GDP (yuan)	PerGDP	10,414	302,669	44,023	31,899	38,968
Population density (person/sq.km)	PopDen	47	41,080	2036	650	5128
Proportion of primary industry in GDP (%)	FirInd	0	0.46	0.14	0.12	0.11
Proportion of tertiary industry in GDP (%)	TerInd	0	0.97	0.39	0.35	0.18
per capita disposable income of urban residents (yuan)	UrbInc	11,647	50,088	24,374	23,159	6589
per capita disposable income of rural households (yuan)	RurInc	4509	27,098	11,235	10,737	4544
Beds in Health Care Institutions per 1000 Persons (unit)	Health	1.52	22.38	4.39	3.24	3.31

## Methods

In the ecological study, the BSTHM was used to map spatio-temporal variation in elderly TB incidence risk factors at the county level. The GeoDetector method was then employed to measure the determinant power of elderly TB socio-economic risk factors and to quantify the interaction effect of these potential risk factors on the disease.

### *Bayesian space–time hierarchy model* (BSTHM)

In the BSTHM, space–time phenomena were broken down into global and local components. The global aspect represented common spatio-temporal variation of TB risk throughout the whole study period and region, whereas the local aspect represented spatio-temporal heterogeneity of the TB risk. The model was used to capture potential space–time interaction factors and to identify spatial hotspots and cold spots of the disease [20, 21].

In this study, new TB cases *y*_*it*_ in county *i* and year *t* were represented as a Poisson distribution, the mathematical expectation of TB case was expressed as the produce between population (*n*_*it*_) and the relative risk (RR) of elderly TB incidence (*r*_*it*_):

1 $$ {y}_{it}\sim Possion\left({n}_{it}{r}_{it}\right) $$

A log link regression function was applied to assess spatio-temporal variation in elderly TB incidence in the BSTHM model [22]. The function was expressed as:

2 $$ \log \left({\mathrm{r}}_{\mathrm{it}}\right)=\alpha +{s}_i+\left({b}_0{t}^{\ast }+{v}_{\mathrm{t}}\right)+{b}_i{t}^{\ast }+{\varepsilon}_{\mathrm{it}} $$

where *α* is a constant term and *s*_*i*_ is a spatial term expressing the spatial distribution of elderly TB risk factors across each county in the study region throughout the whole study period. This spatial term represents spatial heterogeneity in elderly TB incidence risk, and its posterior estimated *exp* (*s*_*i*_) describes the spatial risk of elderly TB in county *i* relative to that in the whole region. *b*_*0*_t* + *v*_*t*_ is a temporal term, expressing the temporal distribution of elderly TB risk factors across each year of the study period in the whole region. Its posterior estimation *exp* (*b*_*0*_t* + *v*_*t*_) describes the overall temporal RR in the study region, t* describes the the middle of the years range, and *v*_*t*_ represents Gaussian noise, indicating a random time effect. The parameter *b*_*i*_ is the local trend in county *i* and represents the spatial heterogeneity of a temporal trend, expressing the spatio-temporal interaction effect. It can be used to measure deviation from common spatio-temporal variation. If the value of *b*_*i*_ is greater than 1, this It indicates that county *i* shows a stronger temporal trend compared with the overall trend of the whole region and vice versa. The term *ɛ*_*1i*_ represents variation not yet explained by the model. In the study, it is assumed to have a Gaussian distribution.

Based on the posterior estimated parameters of the BSTHM, a two-stage method was used to identify spatial heterogeneity in elderly TB RR and to analyse local temporal trends. Spatial RR was first stratified into three classes: hotspot, cold spot or not hot/cold spot, based on the posterior probability *p (exp (si) > 1 | data)*. If the probability of RR in a county was greater than 0.8, it was defined as a hotspot; if the probability of RR in a county was less than 0.2, it was defined as a cold spot; if the probability of RR in a county was between 0.2 and 0.8, the county was defined as neither a hotspot nor a cold spot. Local temporal trends in each spot-class region were then further analysed. In each county, the temporal trend *b*_*1i*_ was stratified into three classes (faster, slower or stable) compared with the overall trend (*b*_*0*_t* + *v*_*t*_) based on the posterior probability *p(b*_*1i*_ *> 0*|*h*_*i*_*, data)*. Regions where temporal trends were decreasing faster had a probability greater than 0.8, regions where temporal trends were decreasing slower had a probability less than 0.2, and regions where temporal trends were stable had a probability of between 0.2 and 0.8. The BSTHM was implemented in WinBUGS software [23].

### GeoDetector q statistic

Recent advances in the *q* statistic of GeoDetector have facilitated investigations of spatial non-linear associations in spatial-temporal data. GeoDetector is a spatial variance analysis method, which can be used to assess the power of an effect factor and analyse the nature of the interactive between a pair of factors. The core concept of the method is that if a potential factor is related to a disease, this factor will be linked to the spatio-temporal distribution of the disease [24–26]. Thus far, GeoDetector has been widely used in the field of public health [27, 28].

The *q* statistic of GeoDetector was used to assess spatio-temporal associations between elderly TB and potential risk factors. The GeoDetector *q* statistic was expressed as:

3 $$ {\displaystyle \begin{array}{l}q=1-\frac{\sum \limits_{h=1}^L{N}_h{\sigma}_h^2}{N{\sigma}^2}\\ {}{\sigma}^2=\frac{1}{N}\sum \limits_{i=1}^N{\left({R}_i-\overline{R}\right)}^2\\ {}{\sigma_h}^2=\frac{1}{N}\sum \limits_{j=1}^{N_h}{\left({R}_{h,j}-{\overline{R}}_h\right)}^2\end{array}} $$

where *N* and *N*_*h*_ are the number of counties in the study region and in the *h*-th stratum (*h* = 1, 2, …, *L*), respectively. *σ*^*2*^ represents the variance in TB incidence in all the counties in the study region, and *σ*_*h*_^*2*^ is the stratified variance in TB incidence in all the counties in the *h*-th stratum. The parameter *R*_*i*_ is the TB incidence in *i*-th county, and *R*_*h*_,_*j*_ is the TB incidence in *j*-th county in the *h*-th stratum. $\overline{R}$ and ${\overline{R}}_h$ refer to the average incidence of elderly TB within the region and a specific stratum, respectively.

The *q* statistic represents the non-linear association between a potential factor and elderly TB incidence. The value lies between 0 and 1, with higher values of the *q* statistic indicating higher determinant power of the variable. If *q* = 0, there is no association between elderly TB incidence and the variable. In contrast, if *q* = 1, then elderly TB incidence is completely determined by the variable. In the study, all the factors in Table 1 were included in the model.

## Results

### Spatio-temporal distribution pattern of elderly TB incidence

From 2009 to 2014 in the study region, the average annual TB incidence was 87.6/10^5^, with apparent spatio-temporal heterogeneity. Spatially, the highest incidence (i.e. above 300/10^5^), was found mainly in the northwest and southeast of the study region, whereas the lowest incidence (i.e. below 1/10^5^) was found mainly in the Beijing and Tianjin megalopolis and its surrounding regions. Temporally, the elderly TB incidence in the region decreased gradually, with an incidence of 99.6/10^5^ in 2009 versus 80.4/10^5^ in 2014.

**Fig. 2:**
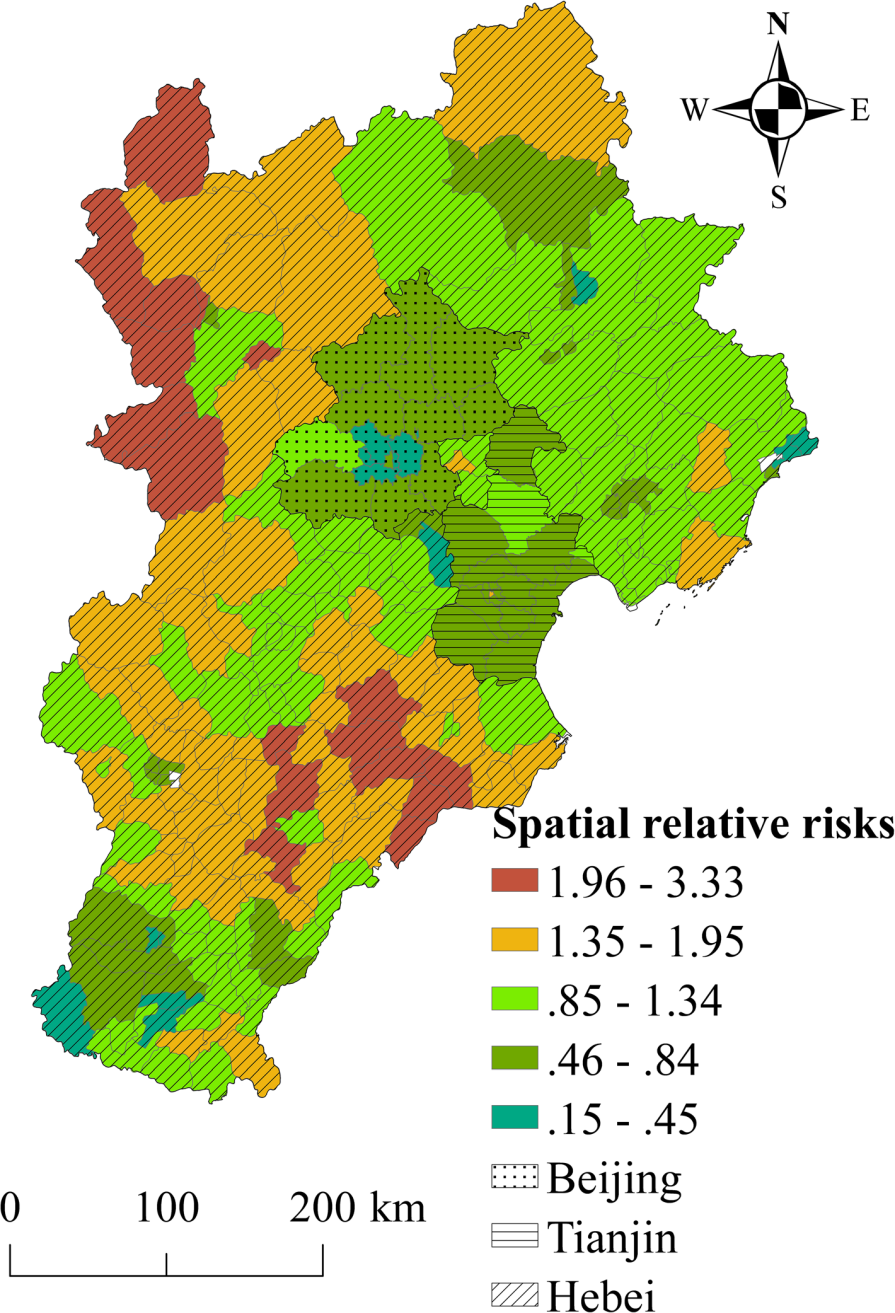
The spatial relative risk of elderly TB incidence in Beijing–Tianjin–Hebei region. This figure was drawn by the authors using ArcGIS 10.2 software

Spatial risk factors for elderly TB incidence were calculated using the BHSTM. The posterior medians of the spatial RRs in the counties, shown in Fig. 2, point to significant heterogeneity in elderly TB incidence risk in the study region. Regions of low elderly TB risk were concentrated mainly in urban areas of Beijing and Tianjin and in counties around this megalopolis. Regions of high TB infection risk included some counties in the northwest mountainous regions and southwest rural regions, far from large cities.

**Fig. 3:**
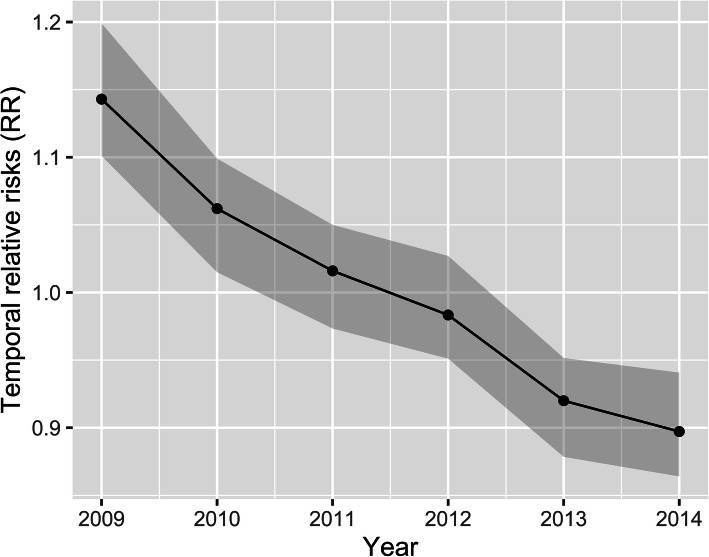
The overall elderly TB incidence RR trend; the posterior medians of exp.(*b*_*0*_t* + v_*t*_) with the 95% confidence interval (*CI*). This figure was drawn by the authors using R (version 4.0.3) software

The common spatial RR of elderly TB incidence was not stable. The results indicated that the RR of elderly TB incidence in all the counties in the study region showed an overall decreasing trend from 2009 to 2014 (Fig. 3). In 2009, the elderly TB temporal RR was 1.15 (confidence interval [CI]:1.10, 1.20) at a significance level of 0.05. In 2014, this index was 0.89 (CI: 0.86, 0.94) at a significance level of 0.05. Although the total overall decreasing trend in the region was rapid, the local downward trend in elderly TB incidence risk varied in different counties.

**Fig. 4:**
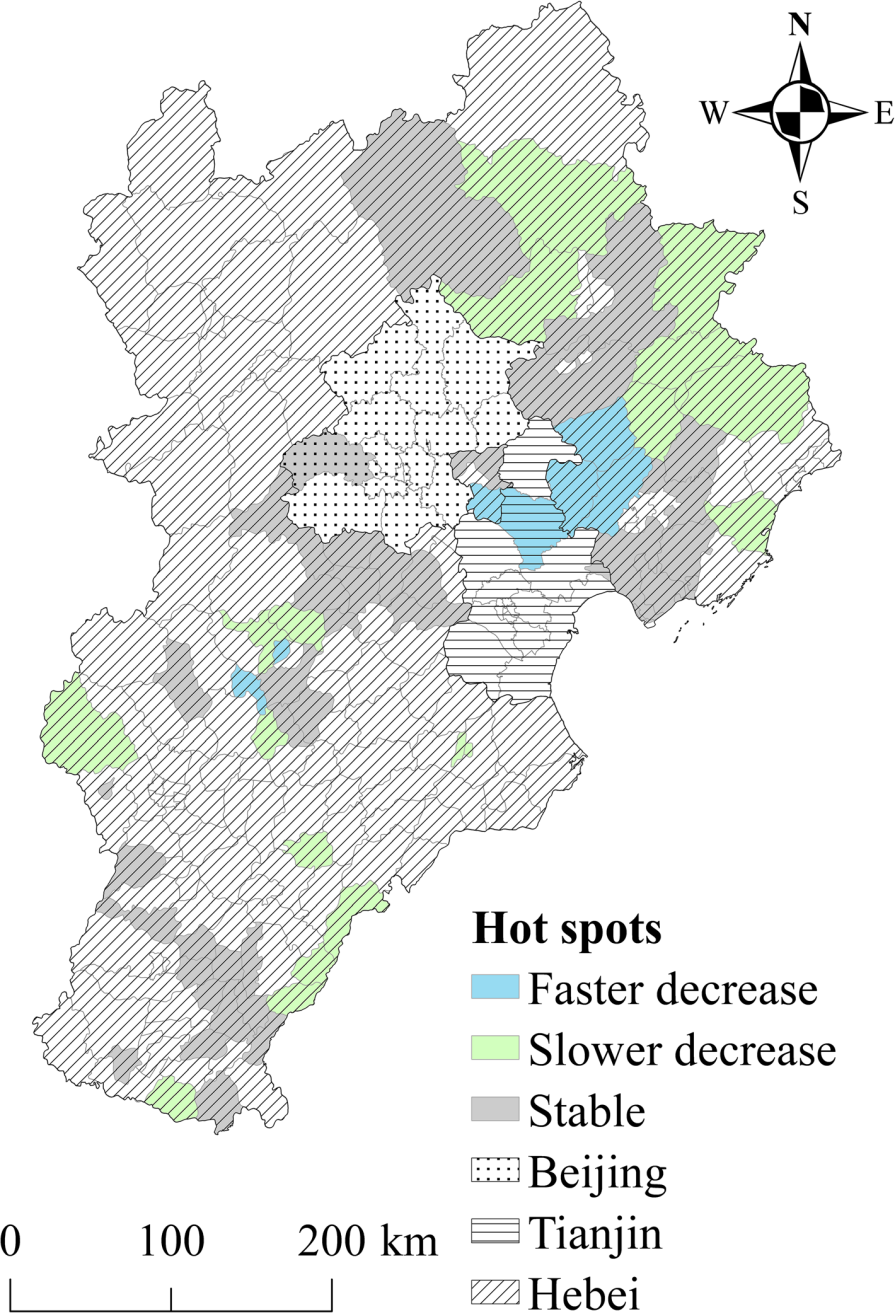
Hotspots with a persistently high risk of elderly TB incidence from 2009 to 2014. This figure was drawn by the authors using ArcGIS 10.2 software

**Fig. 5:**
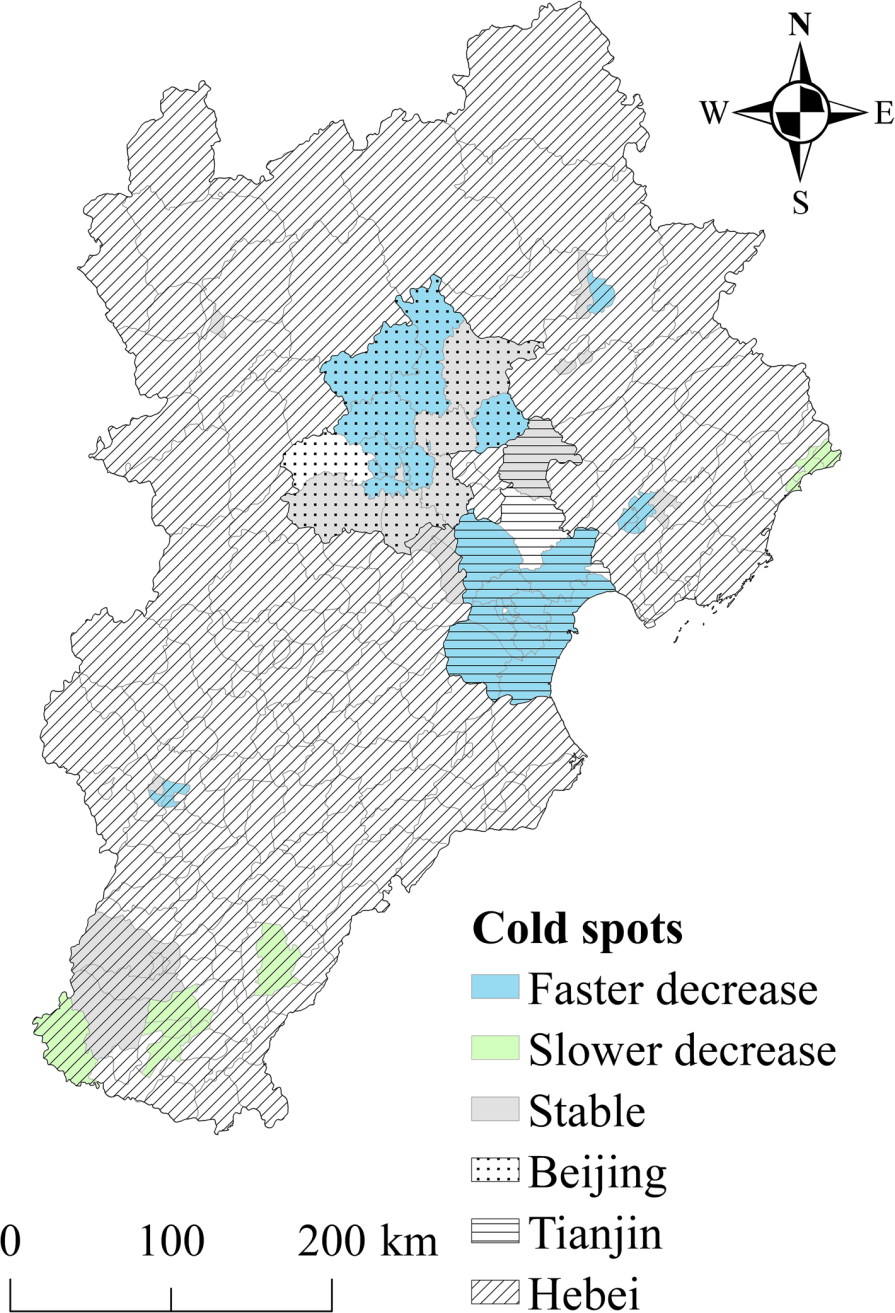
Coldspots with a persistently low risk of elderly TB incidence from 2009 to 2014. This figure was drawn by the authors using ArcGIS 10.2 software

Figures 4 and 5 show the spatial patterns of hot and cold spots of TB incidence in 2009–2014. According to the results, 41.5 and 28% of counties were classified as hotspot and cold spot regions, respectively, with the other 30.5% of counties identified as neither hotspot nor cold spot regions. The counties in hotspots with a high spatial RR value were located primarily in the northwest mountainous regions and southeast rural regions in the study area. Thus, the elderly TB risk was relatively high in these regions. The counties in cold spot areas were located mainly in larger cities and surrounding areas (e.g. the Beijing-Tianjin metropolitan area). These areas had a low spatial RR value, indicating a low level of elderly TB risk.

The local temporal trend of hotspots is shown in Fig. 4. Approximately 26.5% of all hotspot counties showed a faster temporal decreasing trend compared with the overall temporal trend. Thus, these counties may become lower risk areas or even non-hotspots over time. Meanwhile, 18.1% of the hotspot counties showed a slower decreasing temporal trend compared with the overall temporal trend. Over time, there may an increase in the risk of TB cases in these counties due to slower rates of decline in incident cases. In the study, the temporal trend in 55.4% of hotspot counties was consistent with the overall trend. These counties will remain hotspots, with a high risk.

The local temporal trend in cold spots is shown in Fig. 5. In the study, 51.8% of cold spot counties showed a faster temporal decreasing trend compared with the overall temporal trend. Over time, these counties may continue to be cold spots, with a low risk. Meanwhile, 14.3% of cold spot counties showed a slower temporal decrease compared with the overall temporal trend. These counties will likely become higher risk areas or change into hotspots in time. Furthermore, 33.9% of cold spot counties had a temporal trend consistent with the overall trend. These counties will likely remain colds pots, with a low risk.

### Risk factor detection

Elderly TB incidence was selected as the explanatory variable in the *q* statistic calculation. Seven potential socio-economic risk factors were selected as explanatory variables: per capita GDP, population density, proportion of primary industry in GDP, proportion of tertiary industry in GDP, per capita disposable income of urban residents, per capita disposable income of rural households and beds in health care institutions per 1000 persons. Using these variables, the risk factors and their interaction effects on elderly TB incidence were assessed (Table 2).

**Table 2:** The determinant power of single socio-economic factors and their interactive effects on elderly TB incidence

	PerGDP	PopDen	PrimInd	TerInd	UrbInc	RurInc	Health
PerGDP	0.14						
PopDen	0.27	0.16					
PrimInd	0.51	0.23	0.20				
TerInd	0.24	0.19	0.22	0.09			
UrbInc	0.43	0.23	0.23	0.21	0.16		
RurInc	0.46	0.24	0.25	0.24	0.22	0.20	
Health	0.31	0.46	0.54	0.39	0.35	0.39	0.17

All of the values were at 1% level of statistical significance. *PerGDP* Per capita GDP, *PopDen* Population density, *PrimInd* Proportion of primary industry in GDP, *TerInd* Proportion of tertiary industry in GDP, *UrbInc* per capita disposable income of urban residents, *RurInc* per capita disposable income of rural households, *Health* Beds in Health Care Institutions per 1000 Persons

The results showed that socio-economic and health condition factors were the dominant determinants of spatio-temporal variation in elderly TB incidence. Income of rural households showed the strongest association with elderly TB incidence. Low income of rural households was associated with a high incidence of elderly TB incidence, with a *q* value of 0.20 (*p* < 0.01). A large number of beds in health care institutions was associated with a low incidence of elderly TB cases, with a *q* value of 0.17 (*p* < 0.01). Population density, income of urban residents and per capita GDP had relatively low effects on elderly TB incidence, with *q* values of 0.16, 0.16 and 0.14, respectively.

Analysis of interaction effects indicated that health conditions and the proportion of primary industry in GDP had a combined effect on elderly TB risk. The *q* statistic for the interaction was 0.54 (*p* < 0.01). There was also an interaction between health conditions and population density (determinant power: 0.46, *p* < 0.01), health conditions and population density (determinant power: 0.46, *p* < 0.01) and health conditions and rural and urban income (determinant power: 0.39 and 0.35 respectively, *p* < 0.01).

## Discussion

We designed the present study to analyse spatio-temporal variation in TB risk in the over-65 population and to detect potential factors influencing TB risk in a region of unbalanced socio-economic development in China (i.e. Beijing-Tianjin-Hebei). The results revealed the highest risk mainly in rural areas in the northeast and southwest, far from metropolitan areas. Interaction effects between health conditions and socio-economic factors had a significant effect on elderly TB incidence.

In the study, elderly TB risk showed significant spatial heterogeneity, with a relatively high risk primarily in the northwest mountainous regions and south rural regions and a relatively low risk mainly in larger cities and surrounding areas. In terms of risk factors, the proportion of primary industry and tertiary industry in GDP was significantly associated with elderly TB incidence. This finding confirms the impact factors in regions with unbalanced socio-economic development.

Our findings support those of other studies, which reported different urban and rural epidemiological characteristics of elderly TB patients, however, there were inconsistencies between these studies in terms of the TB risk level. For example, in a study carried out in Bangladesh, the authors reported higher TB-related mortality rates in urban than rural regions [29]. Furthermore, results from Tanzania revealed significant differences in the epidemiological characteristics of TB in urban versus rural areas, with recurrent TB in rural but not urban regions [30]. In the present study, we found that elderly TB risk was lower in large cities and their surrounding regions compared with the risk in rural regions. This finding was likely because both health and economic conditions are good in the cities in the study region, with few low-income groups. In contrast, in the study conducted in Bangladesh, regional differences in TB death rates were associated with a high prevalence of the disease in urban areas [29]. These urban areas had a high population density, with one-third of the residents living in slums, a scenario that is favourable for TB transmission [29].

In this study, the regional economic development level was an important index of factors influencing TB transmission. Previous studies revealed an association between TB and per capita GDP, poverty, living standards and under-nutrition [31, 32]. According to a Global Tuberculosis Report 2020 published by the WHO, TB cases begin to decline in some parts of the Western world around the turn of the twentieth century because of improvements in housing, nutrition and economic development [3].

Per capita GDP can provide insights into the effect of in macro-level economic determinants and the effects of policy on spatio-temporal variations in elderly TB incidence. Previous studies indicated that per capita GDP was significantly associated with TB incidence at the country or provincial level [12, 33–35]. A country with a high level of per capita GDP can allocate greater financial resources to TB control. Some reports have provided evidence of a positive association between GDP and the cost of TB case in-treatment. In countries with low socio-economic status and high levels of unemployment, TB inpatient treatment may not be an option for many people for financial reasons. A WHO report indicated that half of all TB patients faced catastrophically high treatment costs [3]. The findings of the present study suggested that unbalanced regional economic development contributes to spatial variation in TB incidence in the study area.

According to many previous studies, income level is an important determinant of TB transmission, with TB closely associated with poverty and an increase in the proportion of TB cases in accordance with low income [3]. Low income may increase TB transmission rates via a direct interaction with poor living conditions and poor nutrition. In this study, income level was a significant predictor of regional-level variation in elderly TB incidence, with the level of rural income having more of an influence on elderly TB incidence than the level of urban income. According to previous studies, regional income imbalances create disparities in access to medical services, as well as delays in diagnoses and medical treatments [3, 36]. Thus, improving the income levels of metropolitan migrant workers and rural residents would be a powerful strategy to control TB among low-income groups.

In this study, population density was significantly associated with elderly TB incidence. Some previous studies reported a high TB incidence in areas of high population density [12, 37]. For example, in a study in Dhaka, Bangladesh, the prevalence of TB in an urban region with a high population density was four times greater than that in other urban areas with lower population densities [38]. Similarly, a study in South Africa showed that the incidence of TB was significantly greater in regions of higher than lower population density [39]. A study in Tanzania reported that crowded living conditions increased TB transmission [39]. However, the present research pointed to a lower incidence of TB among the elderly in the Beijing and Tianjin urban agglomeration area. In this region, populations are concentrated in large cities, with a high level of medical care, good housing conditions and a good welfare system ensuring poverty elimination. Thus, elderly TB incidence in this urban agglomeration area with a high population density was low in our study.

This study has some limitations. TB is a multifactorial disease. Thus, factors other than those considered in the present study (e.g. socio-economic, micro-environmental and personal hygiene habits), may have affected the incidence of TB in the study region. We used aggregate data on a county-level spatial scale in modelling. This may have introduced an ecological fallacy effect for some factors.

Furthermore, new factors, such as epidemic of the other emerging infectious disease, will also influence the transmission of TB. Future research should consider additional factors that account for multi-level relationships in the incidence of TB.

## Conclusions

Our findings shed light on spatio-temporal variation in TB incidence and risk determinants of TB infection among the elderly in a region with disparities in socio-economic development. High-risk hotspots were located mainly in rural areas, far from metropolitan sectors, and medical conditions and economic development level were significantly associated with elderly TB incidence. These variables showed non-linear interactions with each other in influencing TB incidence. TB is of long standing, and the End TB Strategy, adopted by the WHO in 2014, includes targets for reductions in the TB disease burden in 2016–2035 [3], the study depicted a fundamental condition for the control of the disease in regions of unbalanced socio-economic development in the future.

## Data Availability

The TB data the study were from China CDC and were used with their permission. The other data were from statistical yearbook, and they were publicly accessible.

## Abbreviations

TB: Tuberculosis
BSTHM: Bayesian space–time hierarchy model
RR: Relative risk
CI: Confidence interval
